# Strategies of Elicitation to Enhance Bioactive Compound Content in Edible Plant Sprouts: A Bibliometric Study

**DOI:** 10.3390/plants10122759

**Published:** 2021-12-14

**Authors:** María Trinidad Toro, Jaime Ortiz, José Becerra, Nelson Zapata, Paulo Fierro, Marcelo Illanes, María Dolores López

**Affiliations:** 1 Department of Plant Production, Faculty of Agronomy, University of Concepcion, Avenida Vicente Mendez 595, Chillán 3812120, Chile; mariatoro@udec.cl (M.T.T.); nzapata@udec.cl (N.Z.); pfierro@udec.cl (P.F.); marceloillanes@udec.cl (M.I.); 2 Department of Food Science and Chemical Technology, Faculty of Chemical and Pharmaceutical Sciences, University of Chile, Santos Dumont 964, Santiago 8320000, Chile; jaortiz@uchile.cl; 3 Natural Products Chemistry Laboratory, Department of Botany, Faculty of Natural and Oceanographic Sciences, University of Concepción, Víctor Lamas 1290, Concepción 4070386, Chile; jbecerra@udec.cl

**Keywords:** phytochemicals, germinated, antioxidants, elicitation, bibliometric analysis

## Abstract

Vegetable sprouts are a food source that presents high content of bioactive compounds which can also be enhanced through elicitation mechanisms. To better understand the scientific production and research trends on this topic, a bibliometric analysis by means of the Web of Science database was carried out. The results showed significant growth in research on the elicitation of edible plants sprouts. The three most productive journals were the Journal of Agricultural and Food Chemistry, followed by Food Chemistry and LWT-Food Science and Technology. The co-occurrence of keyword analysis of the different authors showed that the main research topics in this domain were ‘germination’, ‘antioxidant activity’, ‘sprouts’, ‘glucosinolates’ and ‘phenolics‘. The countries with the highest number of scientific publications were China, followed by India and USA. The productivity patterns of the authors conformed to Lotka’s law. This study provides an overview of research on elicitation to enrich bioactive compounds in sprouts, and the need to review and update the trends on this subject.

## 1. Introduction

Plants are an indispensable part of our diet since they are a contribution of vitamins or minerals, essentials for a correct physiological function [1]. In recent decades, special attention has been given to a large group of biologically active phytochemicals used in a wide range of industrial applications [2,3].

Synthesis of bioactive compounds is carried out through secondary metabolism, generally at low concentrations (<1% dry weight) and integrated from primary metabolites as common precursors at specific physiological and developmental stages. Therefore, yields of bioactive compounds can be affected by genetic, environmental, agronomic, or geographical factors and impurities [4].

Recently, there is increased knowledge and understanding of the benefits and nutritional contributions provided by the bioactive compounds. However, researchers have gone further and have wondered how to carry out enrichment of bioactive compounds in plants, given that chemical synthesis of secondary metabolites becomes complicated due to the complexity and specificity of their structures, in addition to their high production costs [5].

In addition, currently, there is a great interest in the market of bioactive compounds, such as polyphenols and glucosinolates due to the wide range of uses in the food, pharmaceutical and cosmetic industries. However, obtaining large amounts of phytochemicals to develop new ingredients involves production cost, supply, or seasonality problems and long extraction times. All these factors must be considered to introduce products based on phytochemicals into the market [6]. The cost of polyphenols of winemaking by-products is well known since grape trade dominates the polyphenol market, exceeding USD 700 million in 2015 [7]. Although there are some studies on the economic impact related to essential oils or phenolic compounds, the commercialization of isolated bioactive compounds is still scarce. However, elicitation techniques are a tool to accumulate bioactive compounds in plants obtaining an enriched raw material previously to extraction. Thus, different biotechnological tools have been used to increase the synthesis of secondary metabolites in plants, such as the optimization of culture media, cell cultures, micropropagation, hairy root cultures, elicitation, precursor feeding, or biotransformation. Among all these, the elicitation strategy stands out as a powerful technique to increase bioactive compound synthesis manipulating metabolic pathways [4]. An elicitor can be a chemical substance or physical factor capable of generating defensive morphological and physiological responses that increase or induce the synthesis of bioactive compounds [1,8]. 

Depending on the type of elicitor, the process involves the exogenous application of stressors or inducers, for example, through radiation sources, ultrasound, and foliar irrigation, to the soil or the growing medium [8,9]. This occurs by activating specific receptors in the plant cell membrane and initiating a signaling cascade ending in the expression of genes and transcription factors in the secondary metabolite synthesis pathway, increasing its concentration and accumulation. Thus, the plant is allowed to survive adverse conditions and adapt to the environment [1].

**Figure 1:**
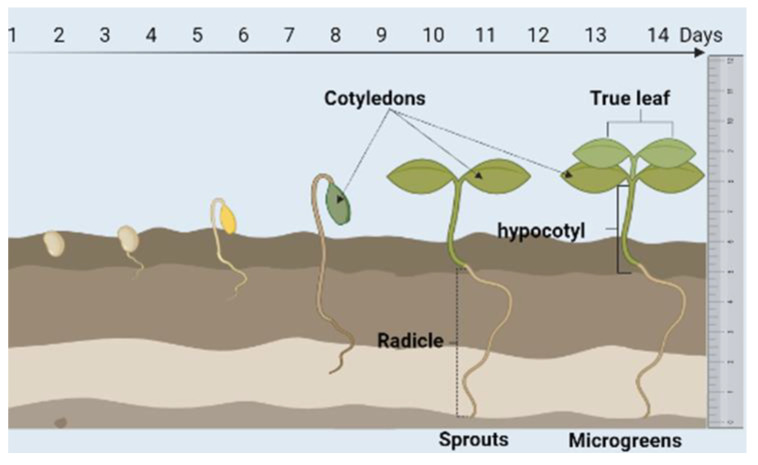
Developmental differences between sprouts and microgreens.

Various studies have shown that sprouts grown from seeds during germination have great nutritional, biological, and medicinal values, evidencing an increase in the antioxidant, antidiabetic, anti-inflammatory, hypolipidemic, and anticarcinogenic activities of plant extracts [10,11,12]. Sprouts and micro-vegetables have higher bioactive compound content than mature plants [1,4], due to the dilution of the phytochemicals resulting from the tissue expansion of a plant in the later development stage [13] (Figure 1). 

Evaluating advances in research on elicitation mechanisms in plant sprouts and how the synthesis of bioactive compounds is induced is crucial to identifying current food-related knowledge. As mentioned, trends in diets rich in bioactive compounds or phytochemicals as sprouts are highlighted. Techniques to enhance these endogenous compounds of plants during the growth period bring us new productive and innovative developments at the agronomic and commercial level because sprouts are characterized by being fast-growing and environmentally friendly. Therefore, these topics have become a priority for discussion by researchers, proposing future strategies to maximize the benefits of these plants (Table 1).

Published research in scientific journals or on websites are used by other researchers for their studies and cited as references in their subsequent articles. This enormous increase in the production of academic publications has generated a large amount of information that can be complex for readers. Therefore, the analysis of the scientific information emerging and past trends leads to the need to address this vast information through mathematical and statistical methods, including bibliometrics analysis [14,15,16,17]. Bibliometrics can be defined as a sub-branch of informetrics, which is aimed to study the status or development of scientific research and the progress of a research field in countries, institutions, research centers, journals, and scientists themselves, detecting changes through patterns and interactions between particles, as well as analysis through a set of mathematical and statistical methods [15,16,18,19]. Since there is no organization and compilation of information regarding the studies on the elicitation mechanisms in sprouts, a bibliometric analysis of the selected publications of the Web of Science (WoS) database was carried out in the present study. This study was aimed to provide information on the basic characteristics of the literature and evaluate the progress and development of scientific research. A brief theoretical approach was first carried out to provide information on the definition of sprouts, ways to identify them, cultivation times, and some examples of different elicitors and their effects on the composition of bioactive compounds. In addition, this approach was followed by a descriptive statistics section. Finally, the bibliometric analysis was carried out, identifying some bibliometric indicators considered of great importance in the evaluation of scientific research, such as productivity analysis of countries, authors, documents and journals, collaboration indexes, and keyword co-occurrence analysis, all of them were analyzed with the support of RStudio software and visualized through the biblioshiny interface. 

Therefore, it is necessary to review and update the research carried out, who directs them, what work networks exist, and what is the focus of the research in everything related to elicitation strategies to enrich edible plant sprouts with bioactive compounds since it is a topic that will continue to be a trend in the next years.

**Table 1:** Biotic and abiotic elicitors used in sprouts and the observed changes in bioactive compound contents after application.

Species	Bioactive Compound	Type of Stress	Dose	Variation ^a^	Authors
Kale	Phenolic Acid	Selenium	0.38 mM	138%	[20]
Yellow maize	Zeaxanthin	NaCl	300 mM	21%	[21]
Radish	Glucosinolates	Methyl Jasmonate	0.25 mM	73%	[22]
Broccoli	Glucosinolates	Sucrose	176 mM	82%	[23]
Wheat	Phenolic Acid	Salix daphnoides extract	1 mL L^−1^	4%	[24]
Peanut	Resveratrol	Ultrasound	240 W	940%	[25]
Lentil	Phenolic content	Mannitol	0.0006 mM	48%	[26]
Buckwheat	Phenolic content	Tyrosine	0.1 mM	30%	[26]
Kidney Bean	Phenolic compounds	Glutamic acid	5 mM	16%	[27]
Lentil	Flavonoids	Phenylalanine	2.410 mg kg^−1^	160%	[28]
Broccoli	Vitamin C	Salicylic acid	0.2 mM	26%	[29]
Mung bean	Antioxidant activity	Oregano extract	1 mL L^−1^	40%	[30]

^a^ Variation is referred as the percentage of increase in the bioactive compound content after elicitation.

## 2. Results and Discussion 

### 2.1. Descriptive Statistics and Exploration of Spatial-Temporal Data

**Figure 2:**
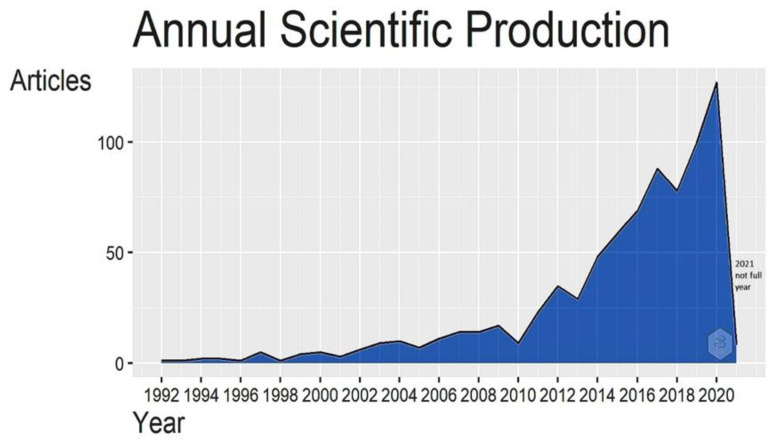
Number of the articles published on elicitation of sprouts in the WoS database between 1992 and 2020.

To describe and provide an overview of the general characteristics of the dataset of the research field, a descriptive analysis was performed. After using the keywords presented in the methodology, a total of 787 studies indexed between 1992 and March 2021 in WoS were selected. During the study period, 2,248 Keywords Plus were identified among 2,738 authors, with an average of 3.48 authors per paper. The average number of coauthors (not first authors) per article was 4.54. A summary of the main information of the related articles is presented in Table S1. The first article in the selected data set was published in 1992 and since then, the research field has had an average annual growth rate of 43% over the 29 years, with an average production of 26.82 articles per year. The number of publications showed a gradual increase with some oscillations. (Figure 2) From 2014 (where there is a leap in the number of publications) until 2020, the total number of articles increased sporadically reaching more than 120 publications, with an average growth rate of 25%. During the first quarter of 2021, 120 articles related to the topic have been published, highlighting the growing interest in the use of elicitors in plant sprouts. The increase in research output on the topic within that period can also be attributed to the need for researchers to find new sources of plant-derived bioactive compounds rapidly and efficiently, given the importance of these compounds in human health. Despite this, safety issues related to bioactive compounds can be controversial due to the great potential they have for rapid auto-oxidation, and to trigger high levels of active enzymes [31].

For example, some authors have mentioned that experimental results demonstrated that elicitors were effective in inducing the production of phytochemicals (phenols, glucosinolates (Figure S1), anthocyanins, etc.) in sprouts, and these compounds were capable of inducing apoptosis in cancer cells [32,33,34].

### 2.2. Bibliometric Analysis

#### 2.2.1. Production Source 

The great advances of this century have caused an evident development of human knowledge, which has been exposed to the world through journals that store the production of researchers. Scientific publication is an essential aspect of the progress or culmination of research, projects, or theses. This acquired knowledge allows the generation of academic exchanges a good communication of knowledge. In addition, it must be useful, precise, valid, novel, and reproducible [35,36].

**Figure 3:**
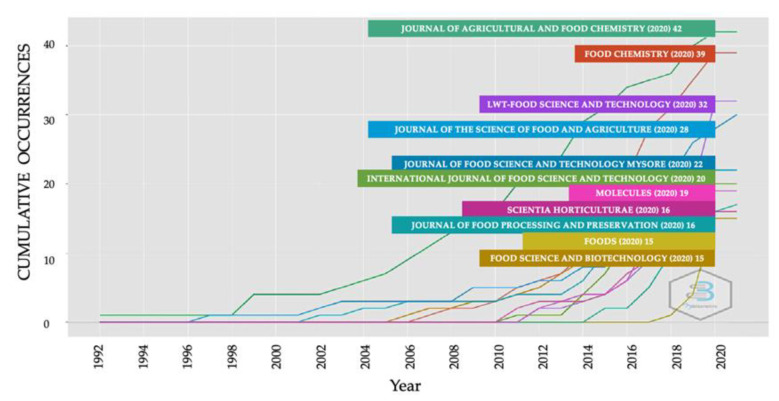
Cumulative occurrences of journals overtime.

The origin and choice of a journal allow readers to rapidly understand the main content and conclusions of the research related in this case to the mechanisms of elicitation in sprouts and bioactive compounds. The articles obtained from the WoS database were distributed across 246 journals in total (Table S2). The top ten journals according to productivity indicators are shown in Table 2. Journal of Agricultural and Food Chemistry (*n* = 42), followed by Food Chemistry (*n* = 39), LWT-Food Science and Technology (*n* = 32), Journal of the Science of Food and Agriculture (*n* = 30) and Journal of Food Science and Technology-Mysore (*n* = 23) were the journals with the highest number of publications on the subject. All of them coincide in their topics related to chemistry and biochemistry in the specific area of food and agriculture, also involving advances in methods and analytical approaches to research. The cumulative number of articles in some journals increased dramatically over time (Figure 3), highlighting the increase produced by the journal of LWT-Food Science and Technology from 2012 where a radical increase in the number of publications was observed. The journals with the most cited articles were Food Chemistry (*n* = 2232), followed by Journal of Agricultural and Food Chemistry (*n* = 1280) LWT-Food Science and Technology (*n* = 399), Journal of the Science of Food and Agriculture (*n* = 393) and Journal of Food Science and Technology-Mysore (*n* = 233). 

#### 2.2.2. Co-occurrence of Author’s Keyword

Keywords symbolize the primary units of research topics in a specific field and the central content of articles. In addition, they facilitate the search for authors and can provide better visualization of the dynamic structure of knowledge. On the other hand, keyword analysis entails a study of the critical points and predictions of future research trends of the most cited articles. Keyword co-occurrence analysis refers to publications in which the same keywords (terms specified by the author) are listed in the same article. With co-occurrence analysis, a network can be mapped. Each node in the network represents a keyword and the link between the nodes represents the co-occurrence of the keywords [37,38,39,40]. 

**Figure 4:**
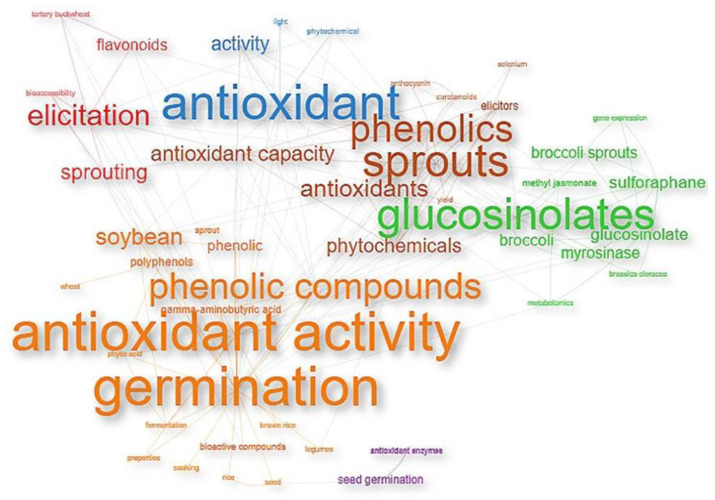
Co-occurrence of author’s keyword manifested on clusters.

The results of the co-occurrence analysis of the top 50 author’s keywords are shown in a network visualization (Figure 4), using Louvain’s clustering algorithm. The author’s keywords have a size proportional to the number of times expressed in terms of the number of occurrences. The colors represent the macro-area group in which the different items are agglomerated and the distance between the keywords is proportional to the relationship between the items. The link between the keywords represents the co-occurrence between them (i.e., keywords that co-occur or occur together), the thickness of the link indicates the occurrence of co-occurrences between keywords (i.e., the number of times keywords co-occur or occur together), the thicker the link between the nodes, the higher the co-occurrences between keywords. 

Among the 2,252 keywords, the first 50 with the highest co-occurrence threshold have been studied. The most numerous clusters with orange color and the largest keyword were ‘germination’ (Figure 4), at the top of the list with *n* = 468 co-occurrences and a high total linkage strength (588.4). It was followed by ‘antioxidant activity’ (*n* = 275), ‘antioxidant’ (*n* = 215), ‘sprouts’ (*n* = 177), ‘glucosinolates’ (*n* = 162), ‘phenolics’ (*n* = 162), ‘phenolic compounds’ (*n* = 120), ‘antioxidants’ (*n* = 116), ‘phytochemicals’ (*n* = 115), ‘sprouting’ (*n* = 111), ‘seed germination’ (*n* = 97), ‘elicitation’ (*n* = 95) ‘soybean’ (*n* = 94), ‘antioxidant capacity’ (*n* = 92), ‘flavonoids’ (*n* = 88), ‘broccoli’ (*n* = 78) ‘glucosinolate’ (*n* = 74), ‘activity’ (*n* = 47), ‘broccoli sprouts’ (*n* = 72) and ‘phenolic’ (*n* = 71). 

This corresponded to a great indicator about the topic in areas related to agronomy, biochemistry, biomedical and food science, and technology.

It is possible to note the connection of the word ‘germination’ with many keywords related to the determination of the antioxidant activity and bioactive compounds of some species, though with less intensity with the word ‘elicitation’ or ‘elicitor’. This is reflected in Figure 4, where the terms are separated by macro-area. This suggests that, although many investigations resort to germination as a technique to obtain plant material, they do not necessarily use elicitors as a mechanism to promote the synthesis of bioactive compounds, (the objectives of their research do not merit it), demonstrating that the sprouts elicitation is a little-explored and fruitful field. It is important to note that a frequent topic studied by most of the articles was related to the determination of the antioxidant activity of sprouts or germinated, in addition to the quantification and determination of bioactive compounds, such as phenols, glucosinolates, or anthocyanins (Table S3). Mainly the growing interest in the determination of the antioxidant qualities of different plant sources has gained more attention from 1995 to the present, due to the known health benefits of consuming fruits and vegetables that contain high amounts of nutrients and phytochemicals [41]. These benefits are related to improved physiological functions or reduced risk of cardiovascular and neurodegenerative diseases, obesity, and cancer [42]. Nowadays, great efforts are being made to improve and enhance the synthesis of these compounds from plant sources. One of them is through elicitation mechanisms. Keywords related to the topic are listed in the same article several times.

**Figure 5:**
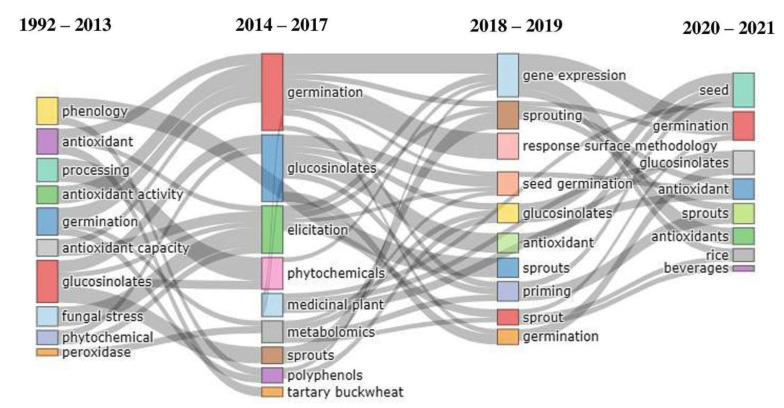
Thematic evolution of the author’s keywords.

Figure 5 shows the evolution of the main author’s keywords and the relationships that appear between them over time. The width of the streamlines is proportional to the number of keywords shared by the connected topics and reflects the degree to which they are related. Here, the new keywords that are changing, such as phytochemicals or glucosinolates are shown. The word ‘elicitation’ begins to be used strongly in the second period, appearing in subsequent periods together with words, such as ‘sprouts’ or ‘seed germination’, the latter being the word with the highest outgoing flow count.

**Figure 6:**
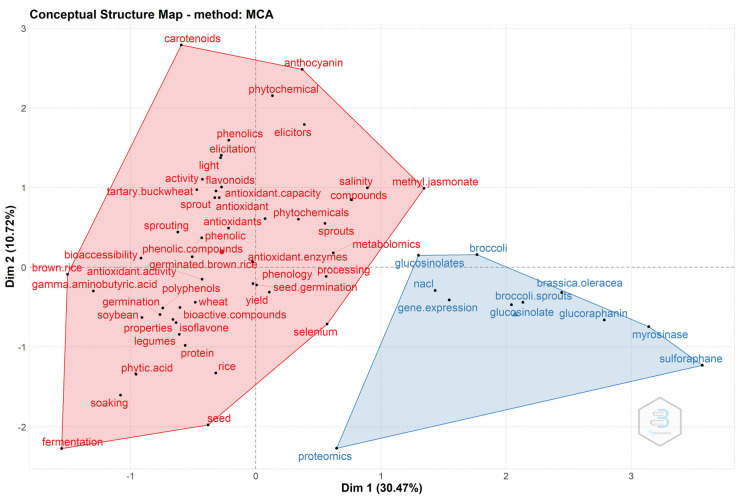
Conceptual structure plot using Multiple Correspondence Analysis (MCA).

In addition, the conceptual structure of the field was mapped using Multiple Correspondence Analysis (MCA) (Figure 6). MCA is a multivariate exploratory technique for the graphic and numerical analysis of multivariate categorical data [43]. A two-dimensional graphic map was prepared with the 50 most relevant author’s keywords, considering the similarities in the distribution of them. The results are interpreted based on the relative positions of the points and their distribution along the dimensions. The more similar these words are and the closer they are to the map, the better they will be represented [43,44]. The broadest cluster corresponds to blue color and represents the keywords belonging to the disciplines of a central theme that have been the object of greater attention and research, though the red cluster shows the areas that have received less investigation. The closer the points are on the chart, the more similar the distribution of keywords will be, involving repeated coexistence in articles. On the other hand, the keywords closest to the central point indicate that they are outstanding in the field of study, whereas the furthest ones are more related to other research topics.

#### 2.2.3. Most Relevant Publications

Imported records represent a collection that can be classified according to the number of local references and citations (LC, within the knowledge domain) and also by the number of global citations (GC, within and outside the knowledge domain) [45]. The top 10 documents in terms of LC and GC are presented in Table 3. The average publication time of these articles was 18 years, where the shortest is 6 years, i.e. Lucía Plaza [46] with 15 LC and 100 GC, and Montserra Dueñas [47] with 14 LC and 49 GC. The most-cited article with 40 LC and 152 GC was from the US researcher Reena Randhir in collaboration with researchers from the same country, published in Process Biochemistry [30], they investigated and evidenced that the phenylpropanoid pathway was stimulated in mung bean sprouts by natural elicitors, such as fish protein hydrolysates, lactoferrin and oregano extract. In addition, they also found that elicitation significantly improved the phenolic, antioxidant and antimicrobial properties of mung bean sprouts. The second most-cited article with 25 LC and 72 GC was international collaborative research conducted by Patricio J Cáceres from Ecuador and Martínez-Villaluenga C, Amigo L and Frias J, these last three Spanish researchers, published in Food Chemistry [48]. They investigated the phytochemical content and antioxidant activity of Ecuadorian brown rice sprouts at different temperatures and hours of darkness. This research group found that germination improved γ-aminobutyric acid, total phenolic compounds, and antioxidant activity in all cultivars. The top 10 most-cited articles agree that elicitation or stress, induced in edible sprouts improves biological activity and boosts the levels of bioactive compounds. These sprouts can be consumed directly as ‘ready to eat food’ or incorporated to staple foods. Moreover, sprouts have been widely used in the prevention of chronic and non-communicable diseases [49,50].

The differences between GC and LC could be the result of the relationship that these articles have with other themes or subjects beyond that of elicitation in edible sprouts, such as the endogenous variations of bioactive compounds (glucosinolates, phenols, etc.) and the implications of the Anti-Tumor-Promoter effect of these compounds. In essence, this shows that Global citation is more related to external themes than internal ones.

#### 2.2.4. Productivity Analysis and Collaboration Networks between Countries

**Figure 7:**
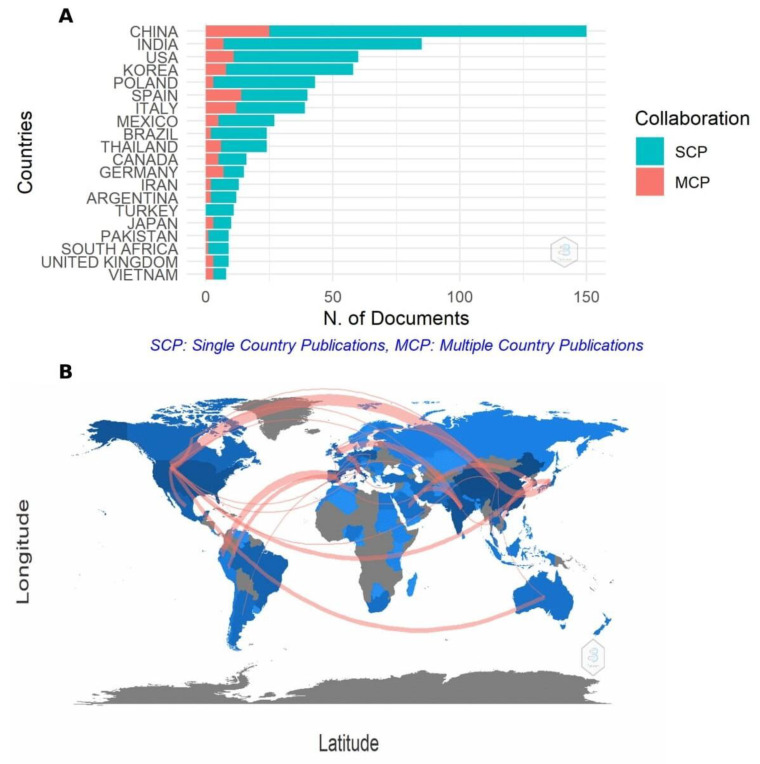
Distribution of the number of publications from single and multiple countries (**A**). Frequency collaboration network between countries working with elicitation in plant sprouts (**B**) (the red links represent the collaborative associations between different countries. The blue color represents the number of publications. The darker the color, the greater the number of publications).

The productivity analysis in terms of the total number of publications per country indicated that China (*n* = 150) is the leading country in terms of the total number of publications, accounting for 19.31% of the total number of articles, followed by India (*n* = 85) and USA (*n* = 60). The list of the 10 most productive countries and international collaborations according to different ranges of MCP (Multiple Country Publications) and SCP (Single Country Publication) is presented in Table S4. The percentage of MCP for China was only 16.7% and in the case of India was 8.2% showing that there is little collaboration between researchers from different countries. However, it is possible to highlight the participation of Spain, where 35% of its publications were in collaboration with other countries. The distribution of countries according to the number of publications produced in collaboration or not with other countries is shown in Figure 7A.

Based on the citation number, the countries with the highest number of publications in the world related to elicitation in sprouts were China (*n* = 2604), followed by the USA (*n* = 2537), and Spain (*n* = 1076).

From the collaboration map (Figure 7B) it can be emphasized that the highest rate of collaboration takes place from the USA to China (10 articles) followed by Spain to Ecuador (six articles) and Japan to China (five articles). Collaborative associations between developed countries and other countries are becoming more frequent, strengthening, and accelerating the rates of progress of developing countries, although these are still low according to the graphs. In addition, it is observed that institutions from developing economies tend to choose co-authors from institutions of international excellence. This suggests that for future research the authors should consider joint research with universities or entrepreneurial institutions in such contexts and increase collaborative networks.

#### 2.2.5. Analysis of Productivity in the Authors

The authors were categorized under different performance indexes. A total of 2,738 researchers authored these 787 articles. Only the 10 most productive authors wrote a total of 123 articles, whereas the rest of the 664 articles were written by other authors.

In general, few authors are responsible for most of the articles and association can be modeled by using Lotka’s Law. Lotka’s Law, one of the basic laws on informetrics was given by Alfred Lotka in 1926. It explains the scientific productivity of authors, which indirectly can help to calculate the publication frequency of researchers (authors) in each area. In simple words, it states "the frequency of publications by authors in a research domain" [51].

**Figure 8:**
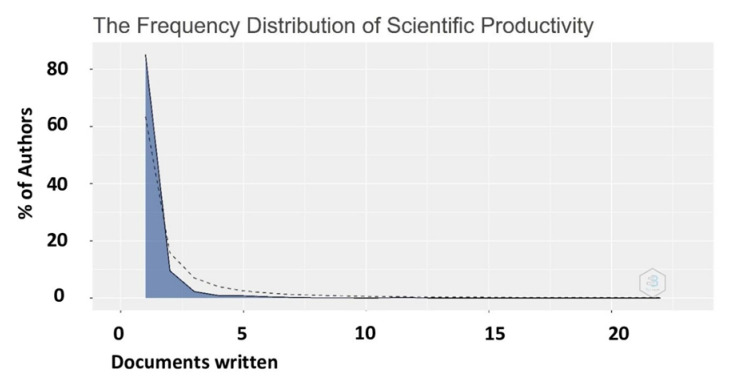
Frequency of authors publishing a given number of articles. The blue area corresponds to the empirical or observed data and the dotted line represents modeled or theoretical relationship according to Lotka’s Law.

The empirical and theoretical frequencies for the total authors who published the selected articles are shown in Figure 8. Consequently, data conform to Lotka’s law. To validate the observation, a beta coefficient β = 2.550, a constant C = 0.424, and a statistical verification with a Kolmogorov–Smirnoff goodness-of-fit test was obtained resulting in 0.911 and a *p*-value of 0.076. Lotka’s Law applied to the productivity of elicitation research in sprouts and the outcome showed how this issue will continue to be a topic of interest. 85.1% of the authors contributed to only 1 article (*n* = 2331), whereas the percentage of authors contributing to two, three and four articles was 9.6% (*n* = 262), 2.3% (*n* = 62) and 0.9% (*n* = 25) respectively. 

According to Van Raan AF [52], several indicators in different aspects of performance to provide a more adequate and multifaceted picture of reality are needed. Therefore, creating an analysis of the scientific performance or productivity of an author in each area of study according to his/her scientific productivity based on the number of publications may not be representative of the overall quality of the researcher. Hence, other indicators are used jointly to evaluate the relevance of an author for the scientific community, such as the impact indicators of publications according to the number of citations and indicators, such as the dominant factor in a defined area [43,53].

Accordingly, the h-index factor can be used to evaluate the productivity and impact of the researcher. The h-index, or Hirsch index [54], indicates the number of publications that have received at least h citations. The number of times a publication is cited is a viable indicator that has been used to represent its influence in a research community and a subject area [55,56].

The h-index is considered a robust, stable, and suitable indicator for assessing productivity [57]. It is easy to calculate and is capable of evaluating researchers with a single number [58]. It has great simplicity of calculation based on the WoS database, so it has been accepted and admitted by the scientific community [59]. In addition, the h-index evaluates the relevance of publications over time [57] through the articles published and the citations received by the articles. Therefore, the values of h increase over time [59]. However, the h-index has certain disadvantages, mainly from its inability to discriminate between active and inactive (or retired) scientists and its weakness in distinguishing between papers that were significant in the past and papers that are "trendy" today [60]. Therefore, it is evident that the h-index of a scientist depends on the scientific age of the author [61]. Moreover, it is also inappropriate when comparing authors from different research fields [62].

Since the h-index factor is susceptible to some limitations, evaluations are often supplemented with g-index, m-index, and other varieties of h-index. The g-index proposed by Egghe [60,63], considers the performance of the top articles [64]. Thus, the m-index is a variant of the h-index and is defined as the h-index of an individual divided by the number of years since his or her first publication [65].

Another way to calculate scientific productivity is according to the dominance factor. The dominance factor (DF) is a ratio that indicates the fraction of articles by several authors in which an academic appears as the first author 

Since the main authors have contributed the maximum work in the selected area, their works have been reviewed to understand the dimensions and trends of the field, taking into account the general results according to the different productivity evaluation criteria. 

The main contributions of the most influential first author in co-authoring with the second most influential author deal with the effects of elicitation on the main health-promoting compounds. The results of one of his articles showed that the phenolic content and the antioxidant potential of lentil sprouts can be improved by treating them under abiotic stress conditions using UV-B as an elicitor, without having any negative influence on the nutritional quality of the lentil sprouts [66]. Other biotic elicitors used by the author were H_2_O_2_, mannitol, NaCl, arachidonic acid, jasmonic acid, and abscisic acid [26]. Other more influential authors investigated the effects of CaCl_2_ on glucosinolate metabolism and isothiocyanate formation, as well as the antioxidant capacity of broccoli sprouts. The results showed that the treatment with CaCl_2_ increased the biosynthesis of glucosinolates, as well as promoted the gene expression of myrosinase, triggering an increased formation of isothiocyanates. As a consequence, an increase in the antioxidant capacity of the sprouts was observed [67]. In another of their articles, they evaluated the effect of growth temperature on glucoraphanin and sulforaphane activity in broccoli sprouts. The results established that the sprouts grown at 25 °C had higher glucoraphanin content and sulforaphane formation than those grown at 20 °C and 30 °C [68]. The effect of glucosinolate activation in sprouts depends on the effects of activation of genes that enable glucosinolate and myrosinase biosynthesis [8].

Finally, several authors have documented the efficacy and importance of elicitation in sprouts to enhance secondary metabolites biosynthesis and accumulation. For instance, the effectiveness of the use of UV-B radiation as an elicitor in the accumulation of vitamin C, phenolic compounds, and flavonoids in mung bean (*Vigna radiata*) sprouts was shown [41]. The increase in phenolic compounds could be attributed to de novo synthesis and transformation. Inducers could stimulate the biosynthetic pathway of phenolic compounds by triggering the accumulation of phenolic compounds in sprouts, through the participation of the enzyme phenylalanine ammonialase (PAL) [8]. 

Other authors determined that sucrose was an effective elicitor to induce the synthesis and accumulation of glucosinolates, phenolic compounds, flavonoids, anthocyanins, and vitamin C in broccoli (*Brassica oleracea L*. var. *botrytis* subvar. *cymosa*) sprouts [23]. On the other hand, it was found that an ultrasound treatment combined with germination can be an effective method of producing resveratrol-enriched peanut sprouts [25].

Consequently, elicitation emerges as a powerful tool to increase and accumulate both primary essential and secondary metabolites with high protective potential for the plant, also important for humans, since these molecules can act by reducing the oxidative stress state responsible for triggering chronic degenerative diseases. In addition, it is environmentally friendly, allows reducing transgenic technology, and is an alternative to conventional cultivation techniques, whose purpose is to enhance the synthesis of phytochemicals with protective effects for health and improving bioactivity in edible plants [22,26].

#### 2.2.6. Limitations and Future Perspectives

The present research gives us a new insight into mechanisms of elicitation in edible sprouts, providing an overview of evolutionary thematic directions and research trends, however, there are some limitations. The literature used provides a wide range of research and topics on scientific articles published in the last three decades and indexed in WoS, although other scientific databases could be revised, such as Scopus and Google Scholar. 

The effect of elicitation on changes in the concentrations of bioactive compounds needs further investigation, due to the rapid development of omics sciences. Hence it could be possible to apply robust and novel techniques to study the effects of elicitation on bioactive compounds in sprouts and at the same time contribute more information in the elaboration of metabolic networks of inducers in sprouts.

The importance of bioactive compounds is due to the strong relationship between the ingestion of fruits and vegetables rich in these compounds and the decreased risk of developing chronic degenerative diseases (e.g., diabetes, neurodegenerative diseases, cancer, etc.). However, other investigations are needed for understanding how foods confer beneficial effects and the complexity developed by the exposome itself and by the interaction of human metabolism and microbiota. 

The optimal conditions of elicitors for each type of sprouts and bioactive compound require novel studies due to the specificity between inducers and sprouts. In addition, possible synergistic effects for these compounds can be analyzed by data analysis techniques and the use of AI-based algorithms.

## 3. Materials and Methods

### 3.1. Theoretical Framework for the Inclusion or Exclusion of Terms

To determine the inclusion or exclusion criteria it is important to previously define some concepts, including the concepts of sprout and microgreens since the terms are often confused. Sprouts are the product obtained from the germination of seeds and are harvested before the development of their true leaves. They can be consumed completely, including their seeds. On the other hand, microgreens are the product of seedlings whose cotyledon and true leaves have fully expanded [69]. Both sprouts and microgreens are in a developmental stage where they contain low amounts of antinutrients. On the contrary, they are rich in nutrients, such as essential amino acids, carbohydrates, and fatty acids from the enzymatic breakdown of macromolecules as well as an elevated amount of bioactive compounds [69,70]. For instance, mung beans can be grown in approximately 5 days obtaining mature sprouts of approximately 5 cm in length. Sprouts up to 8–9 cm long can be grown in 8 days, but longer growth of more than 10 cm should be avoided since they acquire a bitter taste in some species [71]. On the other hand, Brassicaceae sprouts are generally harvested and marketed at 7–8 days of age after germination, considering that this young physiological state is optimal for consumption in terms of biomass and size. It allows manipulation, as well as concentrates a higher content of health-promoting compounds since sprouts have significantly greater concentrations of phytochemicals than mature plants (10–100 times) [72,73,74].

In contrast, it is also important to know and define the concept of elicitor. An elicitor can be a chemical substance or physical factor capable of generating defensive morphological and physiological responses, based on an increase in secondary metabolite synthesis. 

They can be classified according to their nature, in biotic and abiotic elicitors; and according to their origin in exogenous and endogenous elicitors [75]. The first biotic elicitors were described in the early 1970s [76]. The biotic elicitor classification can present an undefined composition, such as crude extracts of yeasts, fungi, and bacteria, and a defined composition, such as purified polysaccharides, glycoproteins, chitosan, or alginate. Among the nature of abiotic elicitor, the variety is wide, for example, salts of metals, such as Ag_2_S_2_O_3_, CuCl_2_, NiSO_4_; osmotic stressors as mannitol, NaCl, KCl, polyvinylpyrrolidone; gases, such as nitric oxide and ethylene; physical stressors like UV radiation, temperature, or drought and internal signaling molecules as jasmonic acid, methyl jasmonate or salicylic acid [4]. On the other hand, elicitors of exogenous origin are found outside the plant cell. Glucomannose, polyamines, polygalacturonase, arachidonic acid belong to this group. Instead, the endogenous origin is formed via secondary reactions induced by biotic or abiotic signals. Hepta-β-glucosides or alginate oligomers belong to this group [75].

### 3.2. Database and Software 

In this section, the methodological techniques and software tools used in this study are explained. For this purpose, a set of publications indexed in Web of Science© (WoS) (March 2021, https://webofknowledge.com/), from Clarivate Analytics related to the investigation of mechanisms of elicitation of secondary metabolite synthesis in sprouts has been collected, refined, processed, and analyzed. WoS covers more than 15,000 journals and more than 90 million documents [77] and allows searching using keywords or co-occurrence analysis of terms [78] tools. 

This bibliometric analysis was developed with the support of RStudio software v.4.0.2, applying a quantitative analysis, structural, performance, and quality indicators [15,79]. RStudio is one of the most widely used tools by researchers, data analysts, and analytical practitioners to perform statistical analyses. R integrates several packages and is updated almost daily, becoming a useful tool for performing meta-analyses, such as bibliometric analyses [80]. Within RStudio, the Bibliometrix 2.2.1 package was applied since it is a useful R tool for the analysis of complete scientific maps and was developed by Aria and Cuccurullo [43]. Recently, the Bibliometrix package has obtained increasing attention from academics and researchers from different areas and disciplines [81,82,83,84]. It provides tools that allow them to perform descriptive analyses from bibliographic data platforms. This package also provides several functions to facilitate the understanding and interpretation of network patterns, including the analysis of the different architectures of a bibliographic collection through conceptual, intellectual, and social structures [15]. The biblioshiny interface was applied, which is interrelated with Bibliometrix, facilitating its main functions, including the size of the labels and the color palette for better legibility of the graphics and images.

### 3.3. Data Collection and Extraction

**Figure 9:**
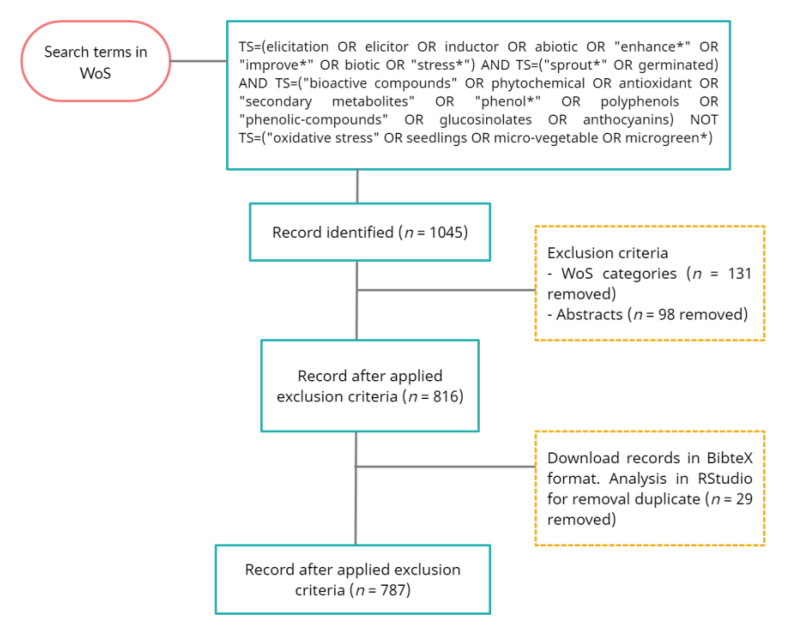
Data collection flow diagram.

The database used to obtain the scientific output information of the present bibliographic review was the WoS. The temporary search period was from inception 1975 to the end of December 2020. Limitations on the year were established to optimize comparability during the bibliometric analysis since the most recent studies have not had time to obtain an appropriate number of citations [53]. This study was carried out by an advanced search in WoS, in all languages and all document types. The searching syntax used was the Boolean operators’ “TS” to indicate de topics, “AND” to link the two fields, and “OR” to combine the building and construction fields in searching the topics, and “NOT” for excluding a topic. Figure 9 provides graphical evidence of the words used in the search and the different phases of the data extraction activity). The citation indexes selected from the WoS main collection were Science Citation Index Expanded (SCI-Expanded), Book Citation Index-Science (BKCI-S) and Emerging Sources Citation Index (ESCI). This first selection included a total of 1,045 articles, filtered to avoid possible inconsistencies or errors. This was performed by identifying thematic areas of interest and a detailed examination of the abstracts to check if the articles identified in the first stage were suitable for analysis subsequently. The articles obtained with a complete record and cited references derived from data collection were exported in BibTeX format for subsequent filtering and analysis, where the duplicate documents were finally eliminated, leaving a final sample of 787 articles.

### 3.4. Evaluation of Scientific Productivity Using Lotka’s law 

To evaluate the distribution of the number of scientific articles published by the various authors in this study, Lotka’s law: $F=c/N^{\beta }\;$was used, which is commonly known as the scientific productivity law. This law describes the frequency F of authors who publish N articles in each field, where β and c are constants. Using the Lotka function, the coefficient β of the bibliographic collection can be estimated and evaluated through a Kolmogorov–Smirnov statistical test whether there is a similarity of this empirical distribution with the theoretical one [43,51]. R package Bibliometrix [43] was used for this analysis.

## 4. Conclusions

In this review, a description and characterization of plant sprouts and bioactive compounds was carried out, specifying how to differentiate them or identify their stage of growth and harvest as well as information on the classification of the different types of elicitors. It is noteworthy that this field of research is interesting and prominent since these enriched and ready-to-eat sprouts can be used in various clinical trials, due to their potential protective and preventive effects against inflammatory and oxidative processes. Secondly, a descriptive analysis of the selected dataset in WoS was performed, followed by a bibliometric analysis, which was carried out during the last 29 years, a period that has allowed generating knowledge and at the same time build a broad vision of many publications on the subject. Most of the selected publications correspond to widely referenced scientific articles. On the other hand, the topic has had a notable increase in the rate of publication in recent years, demonstrating that it is a preponderant and contingent topic. The journals with the highest productivity concerning the total number of articles were the Journal of Agricultural and Food Chemistry, followed by Food Chemistry and LWT-Food Science and Technology, being Food Chemistry the most-cited journal. These journals coincide in their topics related to chemistry and biochemistry in food and agriculture, showing that publications on the subject focus on specific issues rather than broader or more general topics. The research topics were identified from the perspective of keywords, according to the co-occurrence of the author’s keyword analysis. The top five research topics in this domain were ‘germination’, ‘antioxidant activity’, ‘antioxidant’, ‘sprouts’ and ‘glucosinolates’. However, words, such as ‘germination’ and ‘elicitation’ were found with a lower intensity of connection and separated from the macro area. However, this id is not capable to suggest that they were not equally related. This study identifies the most influential publications through the analysis of the Local Citation and Global Citation, which provides unique insight for understanding the development trajectory and intellectual dynamics of the research of the mechanisms of elicitation in edible plants sprouts. The analysis of productivity by country showed that China is the leading country in the total number of publications, followed by India and the USA. However, even though scientific collaboration between countries has increased considerably throughout the 20th century, there are still few collaboration networks. On the other hand, it was found that the productivity patterns of the authors conformed to Lotka’s law, demonstrating that the subject will continue to be of interest, and it is very likely that the number of publications will increase significantly in the coming years. In addition, productivity assessment indicators were used to address different aspects of performance and thus provide a more adequate and multifaceted picture of the reality, allowing to identify the research areas of the main authors. As the world is facing a serious food crisis and over-exploitation of natural supplies because of demographic and economic development, it is necessary greater focus on international collaboration between developed and less developed countries, to create knowledge on new nutritional alternatives. Finally, this study presents new insights into the research of elicitation in plant sprouts and its potential effects in improving the synthesis of bioactive compounds. Moreover, there are significant results of the research executed by the main researchers, which provides justification for future intensive research in aspects of food technology and science, considering its potential to ensure nutrition and the protection of human health.

## Figures and Tables

**Table 2:** Characteristics of the top 10 research journals according to some productivity indicators (h-index, g-index and m-index). TC represents total citations; NP number of publications, TC total citations; and PY_start is years of scientific activity.

Sources	*h-index*	*g-index*	*m-index*	TC	NP	PY_start
Journal of Agricultural and Food Chemistry	22	42	0.7333	2232	42	1992
Food Chemistry	22	35	1.4667	1280	39	2007
LWT-Food Science and Technology	12	19	1.2000	399	32	2012
Journal of the Science of Food and Agriculture	12	19	0.4800	393	30	1997
Journal of Food Science and Technology-Mysore	11	14	0.5500	233	23	2002
International Journal of Food Science and Technology	9	13	0.8182	202	20	2011
Molecules	10	18	1.0000	337	19	2012
Journal of Food Processing and Preservation	5	6	0.7143	57	17	2015
Scientia Horticulturae	9	16	0.8182	304	16	2011
Food Science and Biotechnology	5	10	0.3125	122	15	2006

**Table 3:** The 10 most locally (LC) and globally (GC) cited articles.

1	2004	Randhir R, Lin Y-T, Shetty K.	Stimulation of phenolics, antioxidant and antimicrobial activities in dark germinated mung bean sprouts in response to peptide and phytochemical elicitors.	Process Biochemistry	USA	40	152	Mung vean (*Vigna radiata*); PPP (pentose phosphate pathway); ElicitorsFPH (fish protein hydrosylates); Lactofertinoregano extractG6PDH (glucose-6-phosphate dehydrogenase); GPX (guaiacol peroxidase); Sprouts, Phytochemicals, Peptides; Phenolics, Antioxidants, Antimicrobials, *Helicobacter pylori*.
2	2014	Cáceres PJ, Martínez-Villaluenga C, Amigo L, Frias J.	Maximising the phytochemical content and antioxidant activity of Ecuadorian brown rice sprouts through optimal germination conditions.	Food Chemistry	Ecuador	25	72	Brown rice; Germination; gamma-Aminobutyric acid; Phenolic compounds; Antioxidant activity; Response surface methodology.
3	2012	Lim J-H, Park K-J, Kim B-K, Jeong J-W, Kim H-J.	Effect of salinity stress on phenolic compounds and carotenoids in buckwheat (*Fagopyrum esculentum M*.) sprout.	Food Chemistry	Republic of Korea	23	102	Antioxidant; Buckwheat sprout; Carotenoid; Fagopyrum esculentum; NaCl; Phenolic compound; Salinity stress.
4	2014	Baenas N, García-Viguera C, Moreno DA.	Elicitation: A Tool for Enriching the Bioactive Composition of Foods.	Molecules	Spain	18	118	Elicitor; Phytochemicals; Health; Phenolics; Glucosinolates; Activity.
5	2003	Plaza L, de Ancos B, Cano PM.	Nutritional and health-related compounds in sprouts and seeds of soybean (Glycine max), wheat (*Triticum aestivum.L*) and alfalfa (*Medicago sativa*) treated by a new drying method.	European Food Research and Technology	Spain	15	100	Sprouting; Alfalfa; Soybean; Wheat; Vitamins; Minerals; Phytoestrogens.
6	2004	Kaukovirta-Norja A, Wilhelmson A, Poutanen K.	Germination: a means to improve the functionality of oat.	Agricultural and Food Science	Finland	15	68	Oat; Germination; Processing; Bioactivity.
7	2007	Khattak AB, Zeb A, Bibi N, Khalil SA, Khattak MS.	Influence of germination techniques on phytic acid and polyphenols content of chickpea (*Cicer arietinum L*.) sprouts.	Food Chemistry	Pakistan	15	69	Chickpea; Germination time; Illuminations; Phytic acid; Polyphenols.
8	2014	Świeca M, Sęczyk Ł, Gawlik-Dziki U.	Elicitation and precursor feeding as tools for the improvement of the phenolic content and antioxidant activity of lentil sprouts.	Food Chemistry	Poland	15	34	Elicitation; Bioaccessibility; Precursor feeding; Sprouting; Antioxidant Activity; Phenolic overproduction.
9	2015	Dueñas M, Martínez-Villaluenga C, Limón RI, Peñas E, Frias J	Effect of germination and elicitation on phenolic composition and bioactivity of kidney beans.	Food Research International	Spain	14	49	Kidney beans; Germination; Elicitors; Phenolic compounds; Flavonoids; ACE inhibition activity; Antioxidant activity
10	2006	Fernandez-Orozco R, Piskula MK, Zielinski H, Kozlowska. H, Frias J, Vidal-Valverde C.	Germination as a process to improve the antioxidant capacity of *Lupinus angustifolius L.* var. *Zapaton*.	European Food Research and Technology	Poland	13	58	Lupin; Germination; Antioxidant capacity; Vitamin C; Vitamin E; Polyphenols.

LC: Local citations; GC: Global citations.

## Data Availability

Not applicable.

## Supplementary Materials

The following are available online at https://www.mdpi.com/article/10.3390/plants10122759/s1, Figure S1: Chemical structures of the main glucosinolates, Table S1: Main investigations of elicitation mechanisms in the synthesis of bioactive compounds in sprouts, Table S2: The 246 journals that contain the articles extracted from the database, Table S3: The top 50 author keywords found in the highest number of articles, Table S4: The most productive countries concerning publications on elicitation in sprouts.
