# Supplementary material for: Strategies of Elicitation to Enhance Bioactive Compound Content in Edible Plant Sprouts: A Bibliometric Study
Source: Plants (Basel). 2021 Dec 14;10(12):2759. doi: 10.3390/plants10122759 (PMC8709354; doi:10.3390/plants10122759)
Supplement: Supplementary file 1 [file plants-10-02759-s001.zip › plants-1488471-supplementary.pdf]

Strategies of Elicitation to Enhance Bioactive Compound Content in Edible Plant Sprouts: A Bibliometric Study

María-Trinidad Toro [1], Jaime Ortiz [2], José Becerra [3], Nelson Zapata [1], Paulo Fierro [1], Marcelo Illanes [1] and María-Dolores López [1,*]

Supplementary Materials

Table S1. Main investigations of elicitation mechanisms in the synthesis of bioactive compounds in sprouts.

Description	Results
MAIN INFORMATION ABOUT DATA	
Timespan	1992:2020
Sources (Journals, Books, others)	246
Documents	787
Average years from publication	6
Average citations per documents	16.98
Average citations per year per doc	2.076
References	26,166
DOCUMENT TYPES	
article	722
article; book chapter	7
article; early access	1
article; proceedings paper	10
editorial material	1
meeting abstract	1
review	37
review; book chapter	1
DOCUMENT CONTENTS	
Keywords Plus (ID)	2,248
Author's Keywords (DE)	2,228
AUTHORS	
Authors	2,738
Author Appearances	3,576
Authors of single-authored documents	13
Authors of multi-authored documents	2725
AUTHORS COLLABORATION	
Single-authored documents	17
Documents per Author	0.287
Authors per Document	3.48
Co-Authors per Documents	4.54
Collaboration Index	3.54

Table S2. The 246 journals that contain the articles extracted from the database.

Nº	Sources	Articles
1	JOURNAL OF AGRICULTURAL AND FOOD CHEMISTRY	42
2	FOOD CHEMISTRY	39
3	LWT-FOOD SCIENCE AND TECHNOLOGY	32

4	JOURNAL OF THE SCIENCE OF FOOD AND AGRICULTURE	30
5	JOURNAL OF FOOD SCIENCE AND TECHNOLOGY-MYSORE	23
6	INTERNATIONAL JOURNAL OF FOOD SCIENCE AND TECHNOLOGY	20
7	MOLECULES	19
8	JOURNAL OF FOOD PROCESSING AND PRESERVATION	17
9	SCIENTIA HORTICULTURAE	16
10	FOOD SCIENCE AND BIOTECHNOLOGY	15
11	FOODS	15
12	FRONTIERS IN PLANT SCIENCE	13
13	JOURNAL OF FOOD BIOCHEMISTRY	12
14	ACTA PHYSIOLOGIAE PLANTARUM	11
15	CEREAL CHEMISTRY	10
16	INTERNATIONAL JOURNAL OF MOLECULAR SCIENCES	10
17	JOURNAL OF CEREAL SCIENCE	10
18	JOURNAL OF FOOD MEASUREMENT AND CHARACTERIZATION	10
19	JOURNAL OF FOOD SCIENCE	10
20	JOURNAL OF PLANT PHYSIOLOGY	10
21	FOOD RESEARCH INTERNATIONAL	9
22	HORTSCIENCE	8
23	JOURNAL OF FUNCTIONAL FOODS	8
24	AGRONOMY-BASEL	7
25	ENVIRONMENTAL AND EXPERIMENTAL BOTANY	7
26	FOOD SCIENCE \& NUTRITION	7
27	INDUSTRIAL CROPS AND PRODUCTS	7
28	BMC PLANT BIOLOGY	6
29	TRENDS IN FOOD SCIENCE \& TECHNOLOGY	6
30	ACTA SCIENTIARUM POLONORUM-HORTORUM CULTUS	5
31	EUROPEAN FOOD RESEARCH AND TECHNOLOGY	5
32	FOOD AND BIOPROCESS TECHNOLOGY	5
33	FOOD CONTROL	5
34	HORTICULTURE ENVIRONMENT AND BIOTECHNOLOGY	5
35	INTERNATIONAL FOOD RESEARCH JOURNAL	5
36	JOURNAL OF THE AMERICAN SOCIETY FOR HORTICULTURAL SCIENCE	5
37	PLANT BIOLOGY	5
38	ANNALS OF BOTANY	4
39	CEREAL RESEARCH COMMUNICATIONS	4
40	CYTA-JOURNAL OF FOOD	4
41	FOOD \& FUNCTION	4
42	FOOD BIOSCIENCE	4
43	FOOD SCIENCE AND TECHNOLOGY	4
44	FOOD SCIENCE AND TECHNOLOGY INTERNATIONAL	4
45	HORTICULTURAE	4
46	MOLECULAR NUTRITION \& FOOD RESEARCH	4
47	PLANT CELL AND ENVIRONMENT	4
48	PLANT GROWTH REGULATION	4
49	PLANTS-BASEL	4
50	PROCESS BIOCHEMISTRY	4
51	SOUTH AFRICAN JOURNAL OF BOTANY	4
52	AUSTRALIAN JOURNAL OF BOTANY	3
53	FOOD SCIENCE AND TECHNOLOGY RESEARCH	3
54	GLUCOSINOLATES	3
55	INTERNATIONAL JOURNAL OF FOOD PROPERTIES	3
56	JOURNAL OF CHEMISTRY	3

57	JOURNAL OF ENVIRONMENTAL BIOLOGY	3
58	JOURNAL OF FOOD COMPOSITION AND ANALYSIS	3
59	JOURNAL OF FOOD PROCESS ENGINEERING	3
60	JOURNAL OF PROTEOMICS	3
61	KOREAN JOURNAL FOR FOOD SCIENCE OF ANIMAL RESOURCES	3
62	NOTULAE BOTANICAE HORTI AGROBOTANICI CLUJ-NAPOCA	3
63	PLANT PHYSIOLOGY AND BIOCHEMISTRY	3
64	POSTHARVEST BIOLOGY AND TECHNOLOGY	3
65	SCIENTIFIC REPORTS	3
66	AGRICULTURAL AND FOOD SCIENCE	2
67	BIOLOGIA PLANTARUM	2
68	BIOMED RESEARCH INTERNATIONAL	2
69	BIOSCIENCE RESEARCH	2
70	BOTANY	2
71	CURRENT RESEARCH IN NUTRITION AND FOOD SCIENCE	2
72	EUPHYTICA	2
73	FOOD BIOTECHNOLOGY	2
74	FRESENIUS ENVIRONMENTAL BULLETIN	2
75	FUNCTIONAL FOODS IN HEALTH AND DISEASE	2
76	INTERNATIONAL JOURNAL OF PLANT SCIENCES	2
77	INVASIVE PLANT SCIENCE AND MANAGEMENT	2
78	ITALIAN JOURNAL OF AGRONOMY	2
79	JOURNAL OF APPLIED RESEARCH ON MEDICINAL AND AROMATIC PLANTS	2
80	JOURNAL OF CHROMATOGRAPHY A	2
81	JOURNAL OF EXPERIMENTAL BOTANY	2
82	JOURNAL OF FOOD QUALITY	2
83	JOURNAL OF HORTICULTURAL SCIENCE \& BIOTECHNOLOGY	2
84	JOURNAL OF INTEGRATIVE PLANT BIOLOGY	2
85	JOURNAL OF MICROBIOLOGY BIOTECHNOLOGY AND FOOD SCIENCES	2
86	JOURNAL OF PHYTOPATHOLOGY	2
87	JOURNAL OF PLANT NUTRITION	2
88	NUTRITION \& FOOD SCIENCE	2
89	PAKISTAN JOURNAL OF AGRICULTURAL SCIENCES	2
90	PAKISTAN JOURNAL OF BOTANY	2
91	PHILIPPINE AGRICULTURAL SCIENTIST	2
92	PHYSIOLOGIA PLANTARUM	2
93	PHYTOCHEMISTRY	2
94	PHYTON-ANNALES REI BOTANICAE	2
95	PLANT FOODS FOR HUMAN NUTRITION	2
96	PLANT MOLECULAR BIOLOGY	2
97	PLANTA	2
98	POLISH JOURNAL OF FOOD AND NUTRITION SCIENCES	2
99	QUALITY ASSURANCE AND SAFETY OF CROPS \& FOODS	2
100	RSC ADVANCES	2
101	SEED SCIENCE AND TECHNOLOGY	2
102	SEED SCIENCE RESEARCH	2
103	SPROUTED GRAINS: NUTRITIONAL VALUE PRODUCTION AND APPLICATIONS	2
104	UKRAINIAN FOOD JOURNAL	2
105	ABSTRACTS OF PAPERS OF THE AMERICAN CHEMICAL SOCIETY	1
106	ACTA ALIMENTARIA	1
107	ACTA BIOLOGICA CRACOVIENSIA SERIES BOTANICA	1
108	ACTA BOTANICA BRASILICA	1
109	ACTA SCIENTIARUM POLONORUM-TECHNOLOGIA ALIMENTARIA	1

110	ACTA SOCIETATIS BOTANICORUM POLONIAE	1
111	AFRICAN JOURNAL OF BIOTECHNOLOGY	1
112	AGRICULTURAL RESEARCH	1
113	AGRICULTURAL SYSTEMS	1
114	AGRICULTURE-BASEL	1
115	AGRONOMY JOURNAL	1
116	ALLELOPATHY JOURNAL	1
117	AMERICAN JOURNAL OF POTATO RESEARCH	1
118	ANALYST	1
119	ANALYTICAL BIOCHEMISTRY	1
120	ANALYTICAL CHEMISTRY	1
121	ANNALS OF AGRICULTURAL SCIENCE	1
122	ANNALS OF THE UNIVERSITY DUNAREA DE JOS OF GALATI FASCICLE VI-FOOD TECHNOLOGY	1
123	APPLIED ECOLOGY AND ENVIRONMENTAL RESEARCH	1
124	APPLIED FOOD BIOTECHNOLOGY	1
125	ARCHIVES OF AGRONOMY AND SOIL SCIENCE	1
126	ASIA PACIFIC JOURNAL OF CLINICAL NUTRITION	1
127	AUSTRALIAN JOURNAL OF EXPERIMENTAL AGRICULTURE	1
128	AUSTRALIAN JOURNAL OF PLANT PHYSIOLOGY	1
129	BIOCHIMIE	1
130	BIOLOGIA	1
131	BIOLOGIA FUTURA	1
132	BIOORGANICHESKAYA KHIMIYA	1
133	BIORESOURCE TECHNOLOGY	1
134	BIOSCIENCE JOURNAL	1
135	BIOTECNIA	1
136	BMC GENOMICS	1
137	BOTANICAL SCIENCES	1
138	BOTANICAL STUDIES	1
139	BRAZILIAN ARCHIVES OF BIOLOGY AND TECHNOLOGY	1
140	CANADIAN JOURNAL OF PLANT SCIENCE	1
141	CARPATHIAN JOURNAL OF FOOD SCIENCE AND TECHNOLOGY	1
142	CENTRAL EUROPEAN JOURNAL OF BIOLOGY	1
143	CHEMISTRY CENTRAL JOURNAL	1
144	CIENCIA RURAL	1
145	COGENT FOOD \& AGRICULTURE	1
146	CURRENT TOPICS IN NUTRACEUTICAL RESEARCH	1
147	CZECH JOURNAL OF FOOD SCIENCES	1
148	EGYPTIAN JOURNAL OF BOTANY	1
149	EMIRATES JOURNAL OF FOOD AND AGRICULTURE	1
150	ENVIRONMENTAL PROTECTION STRATEGIES FOR SUSTAINABLE DEVELOP-MENT	1
151	ENVIRONMENTAL SCIENCE AND POLLUTION RESEARCH	1
152	EUROPEAN JOURNAL OF AGRONOMY	1
153	FOOD ANALYTICAL METHODS	1
154	FOOD AUSTRALIA	1
155	FOOD ENGINEERING REVIEWS	1
156	FOOD PACKAGING AND SHELF LIFE	1
157	FOOD PROCESSING: METHODS TECHNIQUES AND TRENDS	1
158	FOOD QUALITY AND SAFETY	1
159	FOOD SECURITY	1
160	FOOD TECHNOLOGY AND BIOTECHNOLOGY	1

161	FREE RADICAL RESEARCH	1
162	FRUITS	1
163	GAYANA BOTANICA	1
164	GENETIC RESOURCES AND CROP EVOLUTION	1
165	GRASAS Y ACEITES	1
166	INDIAN JOURNAL OF EXPERIMENTAL BIOLOGY	1
167	INDIAN JOURNAL OF TRADITIONAL KNOWLEDGE	1
168	INTERNATIONAL JOURNAL OF BIOLOGY AND CHEMISTRY	1
169	INTERNATIONAL JOURNAL OF FOOD SCIENCES AND NUTRITION	1
170	INTERNATIONAL JOURNAL OF GASTRONOMY AND FOOD SCIENCE	1
171	INTERNATIONAL JOURNAL OF PHYTOREMEDIATION	1
172	INTERNATIONAL JOURNAL OF PLANT PRODUCTION	1
173	ISRAEL JOURNAL OF PLANT SCIENCES	1
174	JOURNAL INTERNATIONAL DES SCIENCES DE LA VIGNE ET DU VIN	1
175	JOURNAL OF AGRICULTURAL SCIENCE AND TECHNOLOGY	1
176	JOURNAL OF AGRICULTURAL SCIENCES-TARIM BILIMLERI DERGISI	1
177	JOURNAL OF ANALYTICAL METHODS IN CHEMISTRY	1
178	JOURNAL OF AOAC INTERNATIONAL	1
179	JOURNAL OF APPLIED BOTANY AND FOOD QUALITY	1
180	JOURNAL OF BIOSCIENCES	1
181	JOURNAL OF ESSENTIAL OIL RESEARCH	1
182	JOURNAL OF FOOD AND NUTRITION RESEARCH	1
183	JOURNAL OF FOOD SCIENCE AND TECHNOLOGY-UKRAINE	1
184	JOURNAL OF FUNDAMENTAL AND APPLIED SCIENCES	1
185	JOURNAL OF INTEGRATIVE AGRICULTURE	1
186	JOURNAL OF OLEO SCIENCE	1
187	JOURNAL OF PLANT BIOCHEMISTRY AND BIOTECHNOLOGY	1
188	JOURNAL OF PLANT BIOLOGY	1
189	JOURNAL OF PLANT GROWTH REGULATION	1
190	JOURNAL OF TEXTURE STUDIES	1
191	JOURNAL OF THE CHILEAN CHEMICAL SOCIETY	1
192	JOURNAL OF THE CHINESE INSTITUTE OF ENGINEERS	1
193	JOURNAL OF THE JAPANESE SOCIETY FOR FOOD SCIENCE AND TECHNOLOGY-NIPPON SHOKUHIN KAGAKU KOGAKU KAISHI	1
194	JOURNAL OF ZHEJIANG UNIVERSITY-SCIENCE B	1
195	KOREAN JOURNAL OF HORTICULTURAL SCIENCE \& TECHNOLOGY	1
196	KVASNY PRUMYSL	1
197	LEGUME RESEARCH	1
198	MEAT SCIENCE	1
199	MOLECULAR \& CELLULAR PROTEOMICS	1
200	MOLECULAR PLANT PATHOLOGY	1
201	NUSANTARA BIOSCIENCE	1
202	NUTRIENTS	1
203	OXIDATIVE MEDICINE AND CELLULAR LONGEVITY	1
204	PEERJ	1
205	PESQUISA AGROPECUARIA BRASILEIRA	1
206	PHILIPPINE JOURNAL OF CROP SCIENCE	1
207	PHILOSOPHICAL TRANSACTIONS OF THE ROYAL SOCIETY B-BIOLOGICAL SCIENCES	1
208	PHOTOSYNTHETICA	1
209	PHYSIOLOGY AND MOLECULAR BIOLOGY OF PLANTS	1
210	PHYSIOLOGY INTERNATIONAL	1
211	PHYTON-INTERNATIONAL JOURNAL OF EXPERIMENTAL BOTANY	1

212	PHYTOPATHOLOGY	1
213	PLANT AND SOIL	1
214	PLANT BREEDING	1
215	PLANT CELL TISSUE AND ORGAN CULTURE	1
216	PLANT JOURNAL	1
217	PLANT METHODS	1
218	PLANT PHYSIOLOGY	1
219	PLANT SCIENCE TODAY	1
220	PLOS ONE	1
221	POLISH JOURNAL OF ENVIRONMENTAL STUDIES	1
222	POTATO RESEARCH	1
223	PROCEEDINGS OF THE NATIONAL ACADEMY OF SCIENCES OF THE UNITED STATES OF AMERICA	1
224	PROCESSES	1
225	PROTEOME SCIENCE	1
226	PROTEOMICS	1
227	QUIMICA NOVA	1
228	RESEARCH JOURNAL OF BIOTECHNOLOGY	1
229	REVISTA BRASILEIRA DE CIENCIA DO SOLO	1
230	REVISTA BRASILEIRA DE CIENCIAS AGRARIAS-AGRARIA	1
231	REVISTA BRASILEIRA DE FRUTICULTURA	1
232	REVISTA DE BIOLOGIA TROPICAL	1
233	REVISTA DE LA FACULTAD DE AGRONOMIA DE LA UNIVERSIDAD DEL ZULIA	1
234	REVISTA DE LA FACULTAD DE CIENCIAS AGRARIAS	1
235	REVISTA MEXICANA DE INGENIERIA QUIMICA	1
236	REVISTA VIRTUAL DE QUIMICA	1
237	RICE SCIENCE	1
238	ROMANIAN BIOTECHNOLOGICAL LETTERS	1
239	RUSSIAN JOURNAL OF PLANT PHYSIOLOGY	1
240	SCIENCE OF THE TOTAL ENVIRONMENT	1
241	SEMINA-CIENCIAS AGRARIAS	1
242	SOIL SCIENCE	1
243	SOUTH AFRICAN JOURNAL OF ENOLOGY AND VITICULTURE	1
244	SWEET POTATO: POST HARVEST ASPECTS IN FOOD FEED AND INDUSTRY	1
245	TROPICAL PLANT BIOLOGY	1
246	ZEMDIRBYSTE-AGRICULTURE	1

Table S3. The top 50 author keywords found in the highest number of articles.

N° keywords	Author Keywords	Frequency	%
1	germination	142	14
2	antioxidant activity	84	8
3	antioxidant	53	5
4	glucosinolates	48	5
5	sprouts	45	4
6	phenolics	36	4
7	phenolic compounds	33	3
8	elicitation	27	3
9	antioxidants	25	2
10	sprouting	23	2

11	antioxidant capacity	22	2
12	phytochemicals	22	2
13	soybean	22	2
14	flavonoids	20	2
15	glucosinolate	19	2
16	broccoli sprouts	18	2
17	seed germination	18	2
18	activity	17	2
19	sulforaphane	17	2
20	broccoli	16	2
21	phenolic	15	1
22	myrosinase	14	1
23	bioactive compounds	13	1
24	polyphenols	13	1
25	sprout	13	1
26	antioxidant enzymes	12	1
27	seed	12	1
28	yield	12	1
29	elicitors	11	1
30	gamma-aminobutyric acid	11	1
31	methyl jasmonate	11	1
32	phytic acid	11	1
33	*brassica oleracea*	10	1
34	gene expression	10	1
35	metabolomics	10	1
36	rice	10	1
37	wheat	10	1
38	brown rice	9	1
39	carotenoids	9	1
40	fermentation	9	1
41	legumes	9	1
42	light	9	1
43	phytochemical	9	1
44	properties	9	1
45	selenium	9	1
46	soaking	9	1
47	tartary buckwheat	9	1
48	anthocyanin	8	1
49	bioaccessibility	8	1
50	compounds	8	1

Table S4. The most productive countries concerning publications on elicitation in sprouts.

Rank	Country	Articles	Frequency (%)	SCP	MCP	MCP Ratio (%)
1	China	150	0.1931	125	25	0.1667
2	India	85	0.1094	78	7	0.0824
3	USA	60	0.0772	49	11	0.1833
4	Korea	58	0.0746	50	8	0.1379
5	Poland	43	0.0553	40	3	0.0698
6	Spain	40	0.0515	26	14	0.3500

7	Italy	39	0.0502	27	12	0.3077
8	Mexico	27	0.0347	22	5	0.1852
9	Brazil	24	0.0309	22	2	0.0833
10	Thailand	24	0.0309	18	6	0.2500

Freq: Frequency; SCP: Single Country Publications; MCP: Multiple Country Publications.

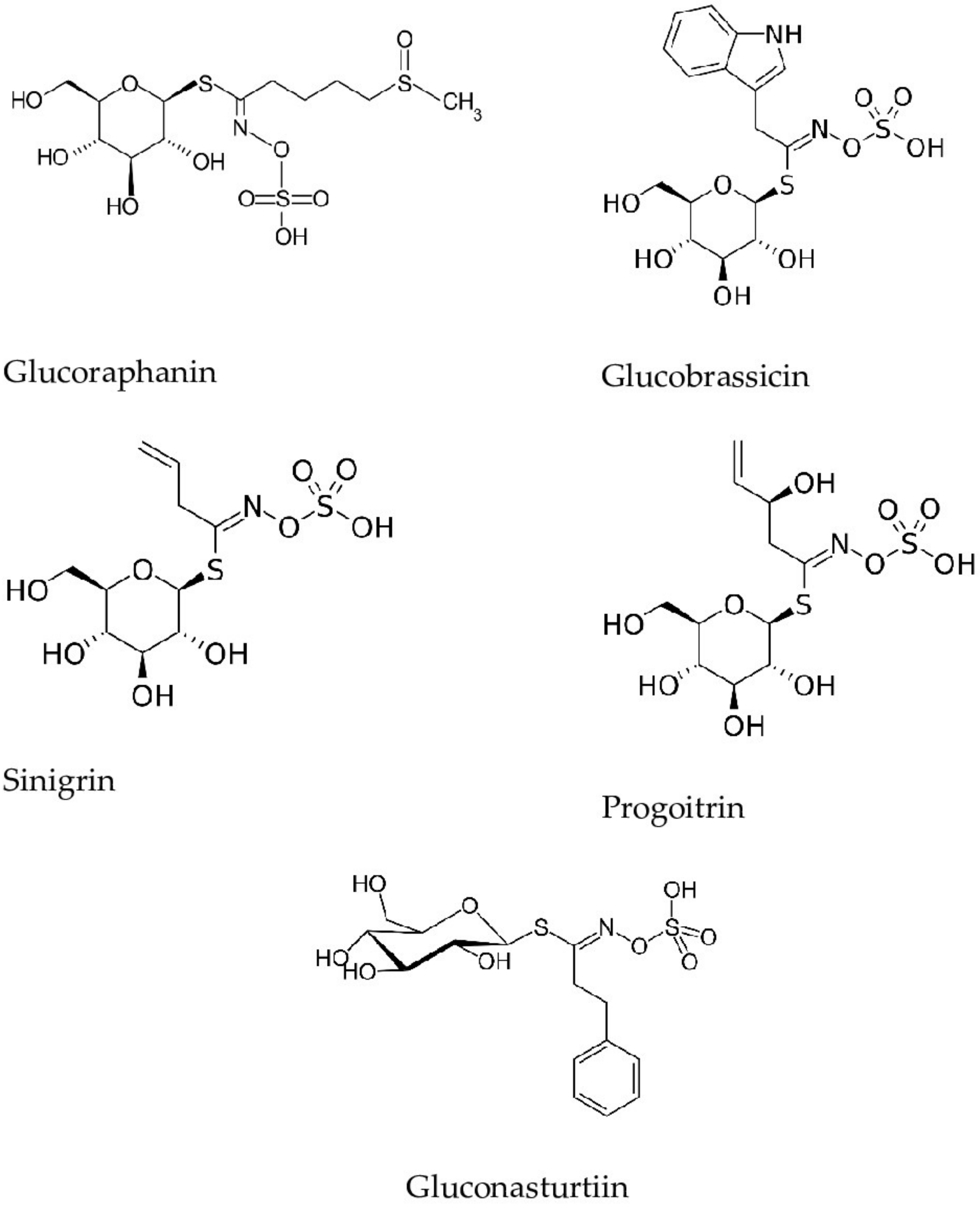

Figure S1. Chemical structures of the main glucosinolates.
